# Effect of Dark Chocolate Extracts on Phorbol 12-Myristate 13-Acetate-Induced Oxidative Burst in Leukocytes Isolated by Normo-Weight and Overweight/Obese Subjects

**DOI:** 10.3389/fnut.2017.00023

**Published:** 2017-06-09

**Authors:** Francesca Ioannone, Giampiero Sacchetti, Mauro Serafini

**Affiliations:** ^1^Faculty of Biosciences and Technologies for Agriculture, Food and Environment, University of Teramo, Teramo, Italy

**Keywords:** chocolate, polyphenols, antioxidant activity, oxidative stress, inflammation, obesity

## Abstract

Oxidative and inflammatory stress represents a major risk factor for cardiovascular disease (CVD) in overweight and obese subjects. Between the different plant foods, chocolate has been shown to decrease CVD risk due to its antioxidant and anti-inflammatory properties. However, as we recently showed in epidemiological studies, meta-analyses, and human trials, dietary antioxidants resulted more effective in subjects characterized by an ongoing oxidative stress, than in healthy people. Aim of this work was to investigate the effect of different concentrations of chocolate phenolic extract (CPE) on *in vitro* free radical production, stimulated by phorbol 12-myristate 13-acetate (PMA), in leukocytes extracted from blood of normo-weight and overweight/obese subjects. Neutrophils from overweight/obese group had a significantly higher free radical production compared to the normo-weight group. In neutrophils, the lowest CPE concentration significantly reduced free radical production in overweight/obese group only, and higher CPE concentrations were effective in both groups. In monocytes, the CPE concentration that was significantly effective in reducing free radical production was lower in overweight/obese subjects than in normo-weight subjects. Chocolate polyphenol extracts inhibit oxidative burst in human neutrophils and monocytes with a higher efficiency in subjects characterized by an unphysiological oxidative/inflammatory stress, such as overweight and obese. Results of this study provide further evidence about a differential role of dietary antioxidant strictly related to the “stress” condition of the subjects.

## Introduction

Oxidative stress, imbalance between reactive oxygen species (ROS) production and the neutralizing capacity of antioxidant mechanism, represents a prepathological status involved in the development of majority of degenerative diseases (1). A raising number of evidence is showing how obesity status is characterized by chronic oxidative and inflammatory stress condition playing a role in the initiation and progression of cardiovascular disease (CVD) (2). Several possible mechanisms that generate oxidative stress in obesity have been identified (3), between them, hyperglycemia (4–6), elevated tissue lipid levels (7, 8), inadequate antioxidant defenses (7, 9), chronic inflammation (10, 11), and endothelial ROS production (12, 13).

The tight link between oxidative and inflammatory stress, in the mechanisms of body’s defenses against stress, is highlighted in the oxidative burst of leukocytes, the innate immune response involving the activation of nicotinamide adenine dinucleotide phosphate (NADPH) oxidase and myeloperoxidase yielding to a massive production of ROS and reactive nitrogen species (14). Polymorphonuclear neutrophils and monocytes are the primary effector cells in the host response to injury and infection. During activation, neutrophils and monocytes generate and release extremely high amounts of ROS through NADPH oxidase system. When there is an excessive and uncontrolled ROS and cytokines production, a condition defined as “low-grade chronic inflammation” takes place and it has been associated with prepathological conditions and degenerative diseases (15, 16). Obesity is associated with an alteration of immune function and a high oxidative burst activity (17). The ROS production upon oxidative burst have been suggested to be modulated by dietary antioxidants that can scavenge free radicals and also exert indirectly their activity by inhibiting enzymes involved in ROS production. Recently, therapeutic tools such as functional foods and nutraceuticals were proposed to treat inflammatory diet-related disease, among which is obesity (18).

Cocoa and, among its principal products, dark chocolate are functional foods that have been shown to display an antioxidant role in humans (19). The antioxidant action of chocolate is due to its high content in flavonoids, such as epicatechin (EC), catechin, and proanthocyanidins, naturally occurring in cacao and cocoa (20) and also to compounds, such as Maillard reaction products, which are formed during cocoa and chocolate processing (21–24). Besides their antioxidant effect, cocoa and dark chocolate have been shown to decrease low-density lipoprotein oxidation and platelet activation, to enhance serum lipid profile, to lower blood pressure, and to promote endothelium-dependent relaxation, supporting the beneficial role of cocoa and dark chocolate in the cardiovascular system (25–30). Cocoa flavonoids has also exhibited promising regulatory effects on immune cells involved in innate and acquired immunity (31) and a recent review pointed out that the immune-modulatory effect of flavonoids is most pronounced in subjects with inflammatory stress than in healthy people (32).

The aim of the present study was to investigate the ability of polyphenol extracts from dark chocolate rich in flavonoids to protect *in vitro* neutrophils and monocytes, isolated from normo-weight and overweight/obese subjects, from oxidative damage and to identify the correlation between body mass indices and oxidative stress.

## Materials and Methods

### Chocolate Samples

Different types of dark chocolate bars rich in polyphenols and having a cocoa percentage between 60 and 70%, identified in previous studies, were analyzed in order to choose the sample with higher antioxidant (i.e., polyphenols) content and antioxidant activity.

### Preparation of Chocolate Extract

Sample extraction was carried out according to the procedure described by Adamson et al. (33). Briefly, chocolate (4 g) was extracted with 25 mL of hexane to remove the lipids. The extract was centrifuged at 2,000 × *g* for 10 min, and the hexane was decanted. Hexane was evaporated at room temperature overnight. The defatted sample was extracted with a solvent mixture (acetone:water:acetic acid, 70:29.5:0.5 v/v/v). After the addition of solvent, the tube was vortexed for 30 s and eventually subjected to sonication at 37°C for 10 min. At the end of extraction, the tube was centrifuged at 2,000 × *g* for 15 min. The supernatant, representing the chocolate phenolic extract (CPE), was collected and used for further analyses.

### Polyphenols Determination

Flavanols and proanthocyanidins determination was carried out by high-performance liquid chromatography (HPLC) analysis according to Ioannone et al. (23). The sample (20 µL) was injected onto a Phenomenex 5 µm normal-phase Luna Silica column, 100 A, 250 mm × 4.6 mm (inside diameter), at 25°C; the column was equipped with a SecurityGuard Cartridges Silica 4 mm × 3.0 mm (inside diameter). Separation of proanthocyanidins was carried out at a flow rate of 1 mL min^−1^ with a linear gradient from A (dichloromethane) to B (methanol) and a constant 4% level of C (acid acetic and water, 1:1 v/v) according to Counet and Collin (34). Gradient elution was as follows: from 14 to 28% B from 0 to 30 min, from 28 to 50% B from 30 to 60 min, from 50 to 86% B from 60 to 65 min, and isocratic from 65 to 70 min. Separation of the compounds was previously made according to retention times by HPLC analysis and then, the compounds were collected according to Counet and Collin (34) and submitted to mass spectrometric (MS) analysis for their identification.

The MS analysis of the HPLC fractions (P1–P10) has been carried out by means of a triple quadrupole mass spectrometer API 2000 from AB-Sciex (Toronto, ON, Canada) equipped with a TurboIon-Spray source. The spectra were acquired by injecting each solution at a flow of 10 μL min^−1^ by a syringe pump; all the analytes were detected in negative ionization with a capillary voltage of −4,500 V, nebulizer gas (air) at 0.21 N mm^−2^, curtain gas (nitrogen) at 0.21 N mm^−2^. The declustering potential was set at −22 V for *m/z* < 1,000 amu; −80 V for *m/z* > 1,000 amu. For the MS/MS experiments, the collision gas was set at 3 (in a scale 0–6) and the collision energy was −20 eV. The spectra were acquired using the AB-Sciex Analyst Software 1.5.

The quantification of single proanthocyanidins was carried out by HPLC analysis using diode array detection. Since proanthocyanidins show a similar absorption coefficient (34), a calibration curve made with (−)-EC was used for their quantification and the results for each proanthocyanidins class were expressed as milligrams of EC equivalents per gram of chocolate. The concentrations of the different classes of phenolic compounds were added to compute the total polyphenol content.

### Total Phenolics Index and Ferric Reducing Antioxidant Power (FRAP) of Chocolate

The total phenolics index (TPI) was determined according to Singleton and Rossi (35). A total of 20 µL of diluted CPE was pipetted into a 96-well plate. A total of 100 µL of Folin–Ciocalteu reagent diluted 1:10 with water and 75 µL of 10% (w/v) sodium carbonate solution were added to each well, and the plate was placed for 2 h at room temperature in dark. Absorbance at 740 nm was then measured using a Sunrise absorbance plate reader (Tecan, Segrate, Italy). Total phenolic index was calculated by a calibration curve, obtained with increasing concentrations of gallic acid, and results were expressed as milligrams of gallic acid equivalents per gram of sample. The reducing power of extracts was measured by the FRAP assay (36), which is based on the reduction of the ferric-tripiridyltriazine (Fe^3+^-TPTZ) complex to the ferrous form at low pH. Briefly, 160 µL of FRAP reagent, prepared daily, was mixed with 30 µL of water and 10 µL of diluted CPE; the absorbance at 595 nm was recorded after a 30 min incubation at 37°C with the Sunrise absorbance plate reader. FRAP values were calculated using a calibration curve obtained with increasing concentrations of Fe^2+^ and expressed as micromoles of Fe^2+^ per micromoles per gram of sample.

### Subjects

Eight normo-weight [four men and four women aged 46 ± 9 years, body mass index (BMI) = 20.54 ± 0.94 kg m^−2^] and seven overweight and obese subjects (four men and three women aged 47 ± 11 years, BMI = 27.21 ± 3.52 kg m^−2^) were recruited for the study. Criteria for inclusion were based on physical examination and BMI. Exclusion criteria are diabetes mellitus, CVD, gastrointestinal tract disease, pulmonary disease, psychiatric disorder, alcohol and drug dependence, history of organ transplant, surgery within 12 months, positive test results for immunodeficiency virus, evidence for hepatitis B virus or hepatitis virus C infection, chronic liver disease or nephropathies, cancer, organ failure, taking lipids-lowering, anti-inflammatory or other medications, taking vitamins, minerals, polyphenols or other supplements, regular consumer of fruits and vegetables of more than four servings per day, following caloric restriction diet, and unbalanced intake of macro and micro nutrients. Waist circumference (WC) was measured in subjects who were categorized as normal or overweight on the BMI scale. All subjects gave written informed consent in accordance with the Declaration of Helsinki.

### Oxidative Burst

Venous peripheral bloods from health volunteers were collected in vacutainer tubes containing ethylene diamine tetraacetic acid (EDTA). All the tubes were stored at room temperature and were immediately used as a source of polymorphonuclear leukocytes (PMNs) for oxidative burst generation assay. PMNs were isolated after bulk erythrocyte lysing. After 10 min of incubation with lysis buffer (1 L of distilled water, 8.02 g of ammonium chloride, 0.84 g of sodium bicarbonate, and 0.37 g of EDTA), the cells were washed twice with phosphate buffer saline (PBS). The method adopted for the measurement of oxidative burst in neutrophils use dihydrorhodamine 123 (DHR123) as probe and phorbol 12-myristate 13-acetate (PMA) for activation (37, 38). DHR123 is an uncharged non-fluorescent probe that passively diffuses across cell membranes and is converted upon oxidation to the fluorescent membrane-impermeant rhodamine 123 (37, 39). Leukocytes (500 cells/μL) staining was performed in PBS with DHR123 (20 µM) for 15 min at 37°C. Leukocytes were split into two different aliquots containing PMA or not (unstimulated sample). Lasting time was 15 min at 37°C, after which cells were stored in ice, to stop reactions, and further rapidly analyzed. Unstimulated cells were used as blank, and trolox was used as standard.

To investigate the *in vitro* ability of dark chocolate to protect neutrophils and monocytes, leukocytes were split into different aliquots containing PMA and different concentration of CPE (previously dried under nitrogen flow and then suspended in dimethyl sulfoxide): 5, 10, 20, 50, and 100 µg mL^−1^. To test whether polyphenols in chocolate extract preparation were responsible for the inhibitory effect of oxidative burst, the effect of EC, one of the major polyphenols in cocoa, was studied at a concentration of 100 µM. Flow cytometric analyses were carried out using a FACScan flow cytometer (BD Biosciences, Milan, Italy). Lymphocytes, monocytes and neutrophils were sorted out using the cytometer by segregating them in gates using forward angle light scatter and side scatter. ROS production was quantified by mean channel fluorescence (MCF) of DHR123 green fluorescence histogram (FL1). Free radicals production (stimulation index) was obtained by dividing the MCF of PMA-stimulated granulocytes by the MCF of unstimulated granulocytes (37).

### Statistical Analyses

All determinations were carried out at least in triplicate. Experimental data are presented as mean values ± SD. Differences between means were compared using the Student’s *t*-test for independent (unpaired) samples. Statistical analyses were performed using the Microsoft Excel software.

## Results

The antioxidant (i.e., polyphenols) content, the total phenolic index, and antioxidant activity of six commercial dark chocolate samples with 60–70% cocoa were determined. Table 1 shows the content of total polyphenols, the total phenolics index, and the FRAP of the selected chocolate samples.

**Table 1:** Total polyphenols content (TPC), total phenolics index (TPI), and ferric reducing antioxidant power (FRAP) of selected chocolate samples.

Sample	TPC (mg g^−1^)	TPI (mg of gallic acid equivalents g^−1^)	FRAP (μmol g^−1^)
Chocolate 1	3.81 ± 0.68	20.34 ± 0.24	213.4 ± 11.5
Chocolate 2	3.38 ± 0.63	19.05 ± 0.16	181.9 ± 42.1
Chocolate 3	3.73 ± 0.63	20.11 ± 0.12	201.2 ± 25.5
Chocolate 4	2.81 ± 0.53	17.33 ± 0.10	150.3 ± 35.4
Chocolate 5	1.41 ± 0.39	12.88 ± 0.11	74.8 ± 22.8
Chocolate 6	2.43 ± 0.52	16.15 ± 0.16	101.4 ± 24.5

Sample 1, showing the highest content of total polyphenols as well as the highest antioxidant capacity, was characterized for its phenolic profile (Table 2) and was used for an *ex vivo* study aimed to investigate the effect of chocolate phenolic extract (CPE) addition on PMA-induced oxidative burst of leukocytes isolated by normo-weight and overweight/obese subjects.

**Table 2:** Monomeric flavanols and proanthocyanidins content of chocolate 1 (mean ± SD).

Compound	mg g^−1^
Epicatechin + catechin	1.57 ± 0.07
Proanthocyanidin dimers	0.70 ± 0.02
Proanthocyanidin trimers	0.44 ± 0.02
Proanthocyanidin tetramers	0.35 ± 0.02
Proanthocyanidin pentamers	0.35 ± 0.01
Proanthocyanidin hexamers	0.14 ± 0.02
Proanthocyanidin heptamers	0.10 ± 0.01
Proanthocyanidin octamers	0.08 ± 0.00
Proanthocyanidin nonamers	0.08 ± 0.00

In order to evaluate relationship between BMI and oxidative stress, the response to PMA for all subjects in both neutrophils and monocytes was first compared. Eventually, the effect of chocolate polyphenols was evaluated by comparing the monocytes and neutrophils activation between the two groups of subjects after adding different concentrations of chocolate extract.

**Figure 1:**
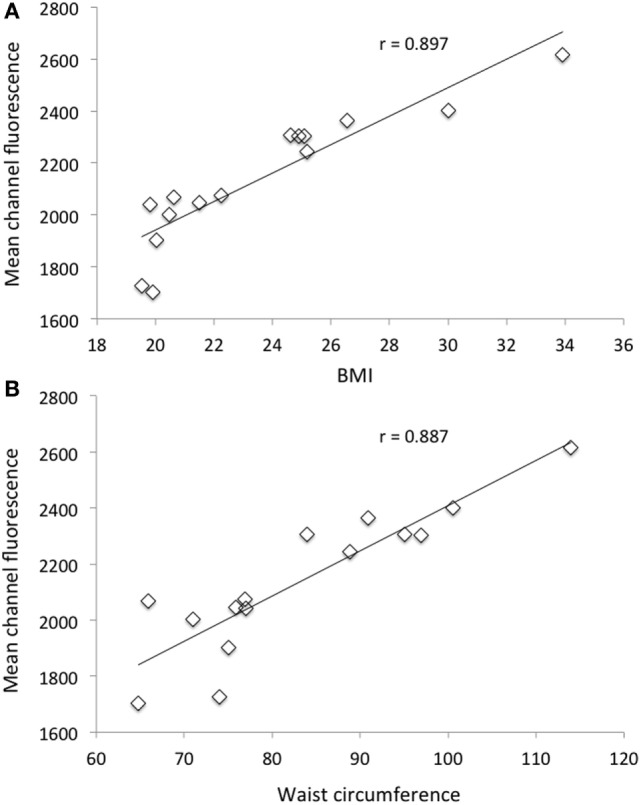
Relationship between mean channel fluorescence (MCF) after phorbol 12-myristate 13-acetate (PMA) stimulation of neutrophils and body mass index **(A)** and MCF after PMA stimulation of neutrophils and waist circumference **(B)** for all selected subjects (*n* = 15).

In Figure 1, the relationship between MCF of neutrophils after PMA stimulation and BMI (Figure 1A) as well as WC (Figure 1B) for all subjects (normo-weight and overweight/obese volunteers) is shown. In both cases, there was a highly significant (*P* < 0.001) positive correlation (*r* = 0.897 and 0.887 for BMI and WC, respectively) between the considered morphometric indices and the response of neutrophils after PMA stimulation. No correlation was found between the same morphometric indices and the response of monocytes after PMA stimulation (data not shown).

**Figure 2:**
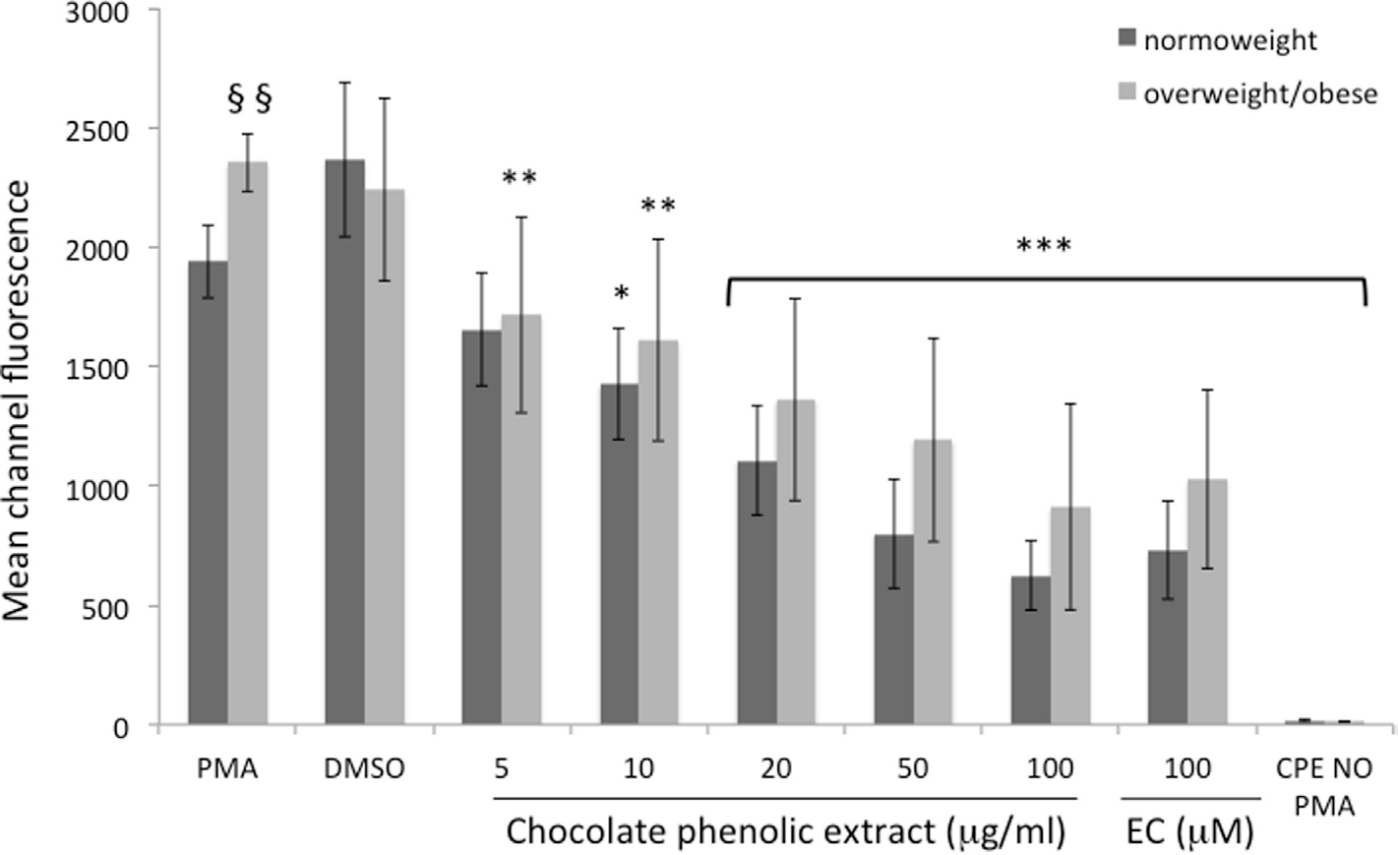
Mean channel fluorescence of neutrophils from normo-weight and overweight/obese subjects in different treatments. Results are expressed as mean ± SD with *n* = 8 for normo-weight and *n* = 7 for overweight/obese groups. *P < 0.05, **P < 0.01, ***P < 0.001: significance of the difference between sample and phorbol 12-myristate 13-acetate (PMA)-stimulated control. ^§§^*P* < 0.05: significance of the difference between PMA normo-weight group and PMA overweight/obese group.

Neutrophils isolated from overweight and obese individuals showed a significantly higher (*P* < 0.05) intracellular ROS generation when compared with those from healthy normo-weight subjects since the mean of fluorescence in normal individuals was 1,954 ± 61 and in overweight/obese ones was 2,353 ± 78 (Figure 2).

In neutrophils, as shown in Figure 2, all the tested concentration of CPE resulted significantly effective in reducing fluorescence of cells of overweight/obese subjects. The lowest cocoa polyphenol concentration (5 µg mL^−1^) significantly reduced fluorescent intensity (*P* < 0.01 vs. PMA) in overweight/obese subjects only, with an inhibition of radical production of 27% respect to PMA. A 10 µg mL^−1^ chocolate concentration fraction showed a significant reduction (*P* < 0.01 vs. PMA) of fluorescent intensity for overweight/obese subjects and a less significant reduction (*P* < 0.05 vs. PMA) in normo-weight subjects (ROS inhibition production respect to PMA 31 and 19%, respectively). Higher chocolate polyphenol concentration (from 20 to 100 µg mL^−1^) showed a more significant reduction (*P* < 0.001 vs. PMA) in fluorescence intensity than low concentration in both groups (65 and 62% for 100 µg mL^−1^, 49 and 55% for 50 µg mL^−1^, and 41 and 37% for 20 µg mL^−1^ for normo-weight and overweight/obese, respectively). EC, used as positive control, exerted a significant (*P* < 0.001 vs. PMA) antioxidant effect in normo-weight and overweight/obese subjects, at a concentration of 100 µM.

**Figure 3:**
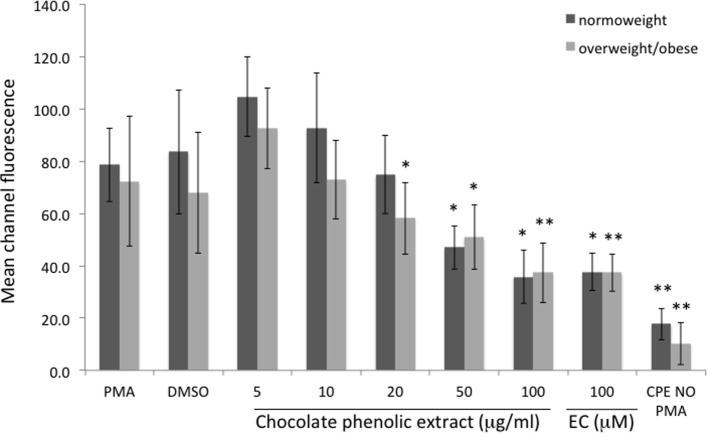
Mean channel fluorescence of monocytes from normo-weight and overweight/obese subjects in different conditions. Results are expressed as mean ± SD with *n* = 8 for normo-weight and *n* = 7 for overweight/obese groups. *P < 0.05, **P < 0.01: significance of the difference between sample and phorbol 12-myristate 13-acetate-stimulated control.

**Figure 4:**
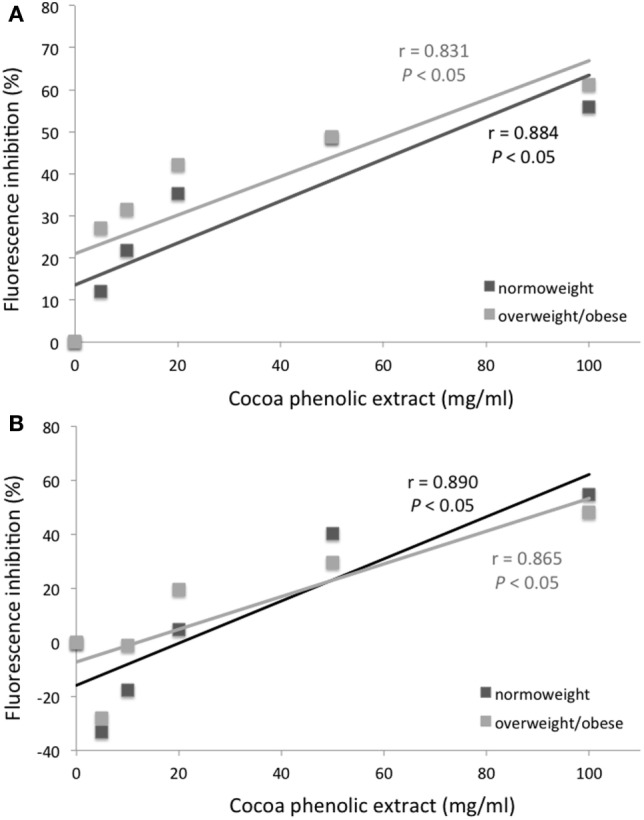
Mean channel fluorescence inhibition (%) of neutrophils **(A)** and monocytes **(B)** from normo-weight and overweight/obese subjects after phorbol 12-myristate 13-acetate stimulation as a function of the concentration of chocolate phenolic extract.

Mean channel fluorescence of monocytes from normo-weight and overweight/obese subjects subjected to PMA-induced burst, in the same experimental conditions previously reported for neutrophils, is shown in Figure 3. A concentration of 100 µg mL^−1^ of chocolate polyphenol extract significantly reduced (*P* < 0.01) fluorescence intensity (MCF = 37 ± 12) in overweight/obese subjects group respect to PMA fluorescence (MCF = 72 ± 25), while in normal weight group, the reduction of fluorescence intensity (MCF = 35 ± 11) with respect to PMA (78 ± 14) was present but less significant (*P* < 0.05). The concentration of 50 µg mL^−1^ resulted the second most effective polyphenol concentration in reducing fluorescence both for normo-weight and overweight/obese subject groups (*P* < 0.05 vs. PMA). At 20 µg mL^−1^ polyphenol concentration, a significant reduction was observed only for overweight/obese subjects (*P* < 0.05 vs. PMA). The lowest chocolate polyphenol concentration (10 and 5 µg mL^−1^) did not show any significant activity in monocytes. EC of 100 µM determined a significant fluorescence reduction for normal weight (*P* < 0.05 vs. PMA) and overweight/obese subjects (*P* < 0.01). A significant correlation was observed between percentage of inhibition of ROS in neutrophils and monocytes and dark chocolate concentrations of phenolics in the normo-weight (Figure 4).

## Discussion

Results of this study clearly indicate that PMA-induced oxidative burst from human neutrophils display a significant correlation with BMI and WCs of the subjects. Neutrophils isolated from overweight and obese individuals showed a higher intracellular ROS generation compared to cells from normo-weight subjects highlighting the presence of oxidative stress associated with excess body fat. We also showed that CPE was effective in reducing the PMA-induced burst of neutrophils and monocytes in a dose-dependent way. Moreover, the inhibitory effect of CPE against ROS production is more evident in leukocytes of subjects characterized by an unphysiological oxidative/inflammatory stress, such as overweight and obese subjects, rather than in normo-weight subjects.

It is known that priming agents able to induce a weak ROS production could enhance the ROS production of neutrophils after exposure to a stimulus that induces an oxidative burst characterized by a strong ROS production (40). Thus, the higher ROS production observed after PMA stimulation of neutrophils from overweight/obese subjects suggest that obesity, similar to other pathological conditions, is associated with an unbalanced redox status, shifting vs. pro-oxidant conditions, which enhance neutrophils response to PMA (41). This suggestion is supported by literature data indicating that obese individuals demonstrate elevated levels of free radicals (42) and a marked decrease in antioxidant defenses (43).

The first evidence of an inhibitory effect of flavonoids on the respiratory burst of neutrophils was by Pagonis et al. (44), who showed that all the studied flavonoids inhibited neutrophils hydrogen peroxide generation. The antioxidant activity of flavonoids against the respiratory burst of neutrophils from healthy human was later confirmed also by other authors (45) displaying a dose-dependent decrease of neutrophil ROS production upon exposure to polyphenol extracts. Cocoa’s flavonoids were proven to moderate oxidative burst derived from a first stimulation of neutrophils with lipopolysaccharide followed by formyl-methionyl-leucyl-phenylalanine aimed to induce strong ROS formation (46). Since the polyphenolic profile of the chocolate used in this study was characterized, the weighed average molecular weight of the phenolics contained in the extract was computed as 786.7 g mol^−1^; thus, the tested concentration of polyphenols was calculated between 6.4 and 127 µM. In neutrophils, the lowest tested concentration (6.4 µM) was effective in reducing significantly ROS production and a 50 µg mL^−1^ (69 µM) chocolate extract determined a protection against ROS formation not significantly different from the 100 µg mL^−1^ (147 µM) catechin positive control. Since the chocolate phenolic extract contains catechin, EC, and polymeric proanthocyanidins, it is possible to affirm that polymeric proanthocyanidins, that are present in a certain amount in chocolate, could show an inhibitory effect of ROS production even higher than catechin.

At low dosages, the CPE was more effective in inhibiting the oxidative burst of neutrophils than that of monocytes. Most of the studies on oxidative burst concern analysis of neutrophils and not monocytes. In fact, these two types of cells differ in structure, time of activation, and behavior during the inflammation response. In a typical acute inflammatory response, there is a well-defined sequence: neutrophils accumulate first and usher monocytes into sites of inflammation where the latter accumulates second (47). Probably, for this reason, neutrophils are most responsive toward the chocolate polyphenol extract than monocytes.

Postprandial stress, arising from the consumption of unbalanced high caloric meals, has been associated with an increased risk for atherosclerosis and obesity (48). Indeed, after the consumption of unbalanced meals, the susceptibility of the organism toward oxidative damage is increased and metabolic and transcriptional pathways are activated leading to a massive increase in the production of free radicals and pro-inflammatory cytokines, through an increase of neutrophil numbers (49, 50). Flavonoid-rich foods have been shown to reduce the onset of oxidative stress derived from postprandial stress condition (51). In this work, we clearly showed, for the first time, that the antioxidant effect of cocoa extract is more evident in neutrophils extracted from subjects characterized by oxidative stress such as overweight and obese. This evidence is in agreement with previous evidences showing a more efficient action of dietary antioxidant when an oxidative stress is present. In a systematic review, Serafini et al. (52) showed that, in dietary intervention studies with plant foods, the percentage of efficiency through an increase of plasma antioxidant defenses was of 58 and of 70%, respectively, on healthy subjects and in subjects characterized by oxidative stress risk factors. Moreover, in the meta-analyses by Lettieri-Barbato et al. (53), the overall action of fruit, vegetables, chocolate, wine, and tea in increasing antioxidant defenses after ingestion was three times higher in subjects with oxidative stress conditions than in healthy subjects. In this study, the marked antioxidant effect of chocolate extracts in neutrophils from overweight and obese subjects suggest that, when a chronic postprandial stress is recurring, leading to excess body fat and to increased neutrophils activation, the presence of exogenous antioxidant might efficiently counteract cellular free radical production, reducing the onset of oxidative stress development.

## Conclusion

Dark chocolate polyphenols showed an inhibitory effect on PMA activated oxidative burst in white blood cells, in a concentration-dependent manner. This inhibitory effect is significantly more evident in subjects characterized by an unphysiological oxidative/inflammatory stress, such as overweight and obese subjects. Although antioxidant capacity of dark chocolate in human is deeply related to the low bioavailability of the flavonoids contained in the foodstuff, our results suggest that chocolate polyphenols might efficiently display a higher antioxidant/anti-inflammatory effect in overweight and obese subjects, characterized by an ongoing oxidative and inflammatory stress, than in normo-weight subjects. Results of this study provide further evidence about a differential role of dietary antioxidant related to the “stress” condition of the subjects. In future human trials, one criteria of selection of the subjects should be the presence of oxidative stress condition in order to maximize the chance of success of the antioxidant intervention. Specifically, more evidences in human trials are needed in order to unravel the role of cocoa and polyphenols in reducing oxidative and inflammatory stress in overweight and obese subjects.

## Ethics Statement

Due to the nature of the study, simple collection of blood specimens, the involvement of ethical committee was not necessary. All subjects remain anonymous and gave written informed consent in accordance with the Declaration of Helsinki.

## Author Contributions

MS designed the experiment; FI and GS conducted the experiments; MS and GS contributed to data analysis and interpretation; FI, GS, and MS contributed to the manuscript drafting.

## Conflict of Interest Statement

The authors declare that the research was conducted in the absence of any commercial or financial relationships that could be construed as a potential conflict of interest.

## Abbreviations

BMI, body mass index; CPE, chocolate phenolic extract; DHR123, dihydrorhodamine 123; EDTA, ethylene diamine tetraacetic acid; FRAP, ferric reducing antioxidant power; GAE, gallic acid equivalent; HPLC, high-performance liquid chromatography; MCF, mean channel fluorescence; MS, mass spectrometry; NADPH, nicotinamide adenine dinucleotide phosphate; PBS, phosphate buffer saline; PMA, phorbol 12-myristate 13-acetate; PMNs, polymorphonuclear leukocytes; ROS, reactive oxygen species; TPC, total polyphenols content; TPI, total phenolics index; WC, waist circumference.
